# Gasdermin D-Mediated Pyroptosis in Diabetic Cardiomyopathy: Molecular Mechanisms and Pharmacological Implications

**DOI:** 10.3390/molecules28237813

**Published:** 2023-11-28

**Authors:** Zhou Liu, Yifan Chen, Yu Mei, Meiling Yan, Haihai Liang

**Affiliations:** 1 Institute of Chinese Medicine, Guangdong Pharmaceutical University, Guangzhou 510006, China; liuzhou0506@outlook.com (Z.L.); my20158@outlook.com (Y.C.); meiyu1117@outlook.com (Y.M.); 2 Key Laboratory of Glucolipid Metabolic Disorder, Ministry of Education, Guangzhou 510006, China; 3 Guangdong TCM Key Laboratory for Metabolic Diseases, Guangzhou 510006, China; 4 Guangdong Metabolic Diseases Research Center of Integrated Chinese and Western Medicine, Guangzhou 510006, China

**Keywords:** pyroptosis, Gasdermin D, diabetic cardiomyopathy, heart, inflammasomes

## Abstract

Diabetic cardiomyopathy (DCM) is a pathophysiological condition triggered by diabetes mellitus (DM), which can lead to heart failure (HF). One of the most important cellular processes associated with DCM is the death of cardiomyocytes. Gasdermin D (GSDMD) plays a key role in mediating pyroptosis, a type of programmed cell death closely associated with inflammasome activation. Recent studies have revealed that pyroptosis is induced during hyperglycemia, which is crucial to the development of DCM. Although the effects of pyroptosis on DCM have been discussed, the relationship between DCM and GSDMD is not fully clarified. Recent studies gave us the impetus for clarifying the meaning of GSDMD in DCM. The purpose of this review is to summarize new and emerging insights, mainly discussing the structures of GSDMD and the mechanism of pore formation, activation pathways, molecular mechanisms of GSDMD-mediated pyroptosis, and the therapeutic potential of GSDMD in DCM. The implications of this review will pave the way for a new therapeutic target in DCM.

## 1. Introduction

Diabetic patients have a high risk of cardiovascular dysfunction, which is a leading cause of death. The underlying pathogenesis of diabetic cardiovascular disease is intricate and may arise from hyperglycemia, dyslipidemia, atherosclerosis, vascular dysfunction, hypertension, and prethrombotic states [1]. In 1972, Shirley Rubler identified a new type of cardiomyopathy in diabetic patients called diabetic cardiomyopathy (DCM) [2], which is defined as structural and functional abnormalities of the heart in patients with diabetes mellitus (DM), with the exception of coronary artery disease, hypertension, and other preexisting cardiovascular conditions [3]. Many factors are involved in the occurrence of DCM, including insulin resistance, hyperglycemia, increased oxidative stress, lipid peroxidation, calcium treatment of cardiomyocytes, mitochondrial dysfunction, endothelial dysfunction, and cell death. With limited regenerative ability, cardiomyocytes could be disrupted by multiple factors, such as oxidative stress, inflammation, and calcium mishandling, enhancing the dysfunction of the systolic system, compensatory cardiac hypertrophy, myocardial fibrosis, and electrocardiographic conduction disturbance [4]. In biology, cell death is broadly classified as necrosis and programmed cell death (PCD). To maintain the normal structure and function of the cardiovascular system, a balance must be maintained between the formation and death of cells present in cardiac tissues. Excessive PCD can result in structural destruction and functional impairment of the cardiovascular system, eventually leading to cardiomyopathy [5]. PCD includes pyroptosis [6], apoptosis [7], ferroptosis [8], autophagy [9], necroptosis [10], cuproptosis [11], and more. PCD can be classified into different forms based on their morphological characteristics. Previous studies have demonstrated that apoptosis is increased in HFD-induced DCM [12], and it is an 85-fold cardiomyocyte apoptosis in the hearts of diabetic patients than that in non-diabetic control groups [13]. Recent studies have reported that AS-IV improves cardiac function and myocardial injury via inhibition of ferroptosis in diabetic rats [14]. More and more evidence has demonstrated that PCD of cardiomyocytes is a major contributor to the development of DCM. However, it is not always detrimental; under certain conditions, it can play a protective role in the heart. For instance, when acute stress is present, autophagy serves as a cardioprotective response. Autophagosome formation and autophagy were reduced in mice with DCM, promoting the apoptotic response in cardiomyocytes [15].

In recent years, with the occurrence of plentiful scientific research, multiple forms of PCD have been studied in depth; among them, pyroptosis is considered one of the crucial factors contributing to the pathological progression of DCM. In 1992, pyroptotic cell death was observed for the first time in macrophages, in which the cell lyses rapidly after being infected with *Shigella flexneri*, a Gram-negative bacterium [16]. In 2001, the term “pyroptosis” was first proposed, which was classified as a form of cell death due to its inflammatory nature and recognized as a proinflammatory PCD that depended on caspase-1 and GSDMD [17]. Pyroptosis is a crucial component of innate immunity, leading to different immune outcomes and low or high levels of inflammatory response. Moreover, an inflammatory response will depend on the cellular environment and cell type where pyroptosis is executed [18]. If the immune or inflammatory response is excessively activated or suppressed, the result can be serious, such as cardiac remodeling in humans [19]. Pyroptosis can occur in cardiomyocytes, cardiac fibroblasts, vascular endothelial cells (VECs), and vascular smooth muscle cells (VSMCs), eventually leading to heart damage. Recently, increasing evidence has shown that alleviating cardiac pyroptosis has a beneficial effect on DCM, and it has been proposed as a strategy for combating DCM. In this review, we summarize and discuss cell pyroptosis mechanisms mediated by GSDMD, which is the most extensively studied pyroptosis-executing protein in DCM, to provide researchers with the new perspective that GSDMD-mediated pyroptosis might be a target for effective treatment of DCM.

## 2. Pyroptosis

Recently, it has been discovered that necroptosis, apoptosis, and pyroptosis are superimposed, and, therefore, a concept of total cell death has been proposed, which is called PANoptosis [20]. It has been reported that activation of ZBP1, which is a nucleic acid innate immune sensor that regulates host defense response, will trigger PANoptosis via activating the RIP3, caspase-8, and NLRP3 pathways [21,22,23]. Interestingly, studies have shown that TRIF-dependent TLRs (i.e., TLR3 and TLR4) induce caspase-8-dependent cleavage of the suppressor of cytokine production, N4BP1, which leads to increased cytokine production and release [24]. TLRs that are dependent on MyD88 fail to target N4BP1, resulting in a reduced cytokine response. However, binding of TNFα to TNFR1, subsequent activation of caspase-8, and cleavage of N4BP1 allowed TRIF-independent TLRs to induce cytokine production. Hence, caspase-8 is an essential link and molecular switch in signal integration that controls PANoptosis [25,26]. Recently, increasing attention has been paid to pyroptosis due to its association with innate immunity and diseases. Similarly to apoptotic or necroptotic cells, pyroptotic cells release a number of intracellular molecules that can activate the immune system by serving as alarmins and ‘find me’ signals. However, unlike apoptosis and necroptosis, pyroptosis is a type of regulated cell death that is critically dependent on the formation of plasma membrane pores, swelling, and cell rupture induced by members of the gasdermin protein family, typically caused by the activation of inflammatory caspases [27], and activation of inactive cytokines like interleukin-1β(IL-1β) and interleukin-18(IL-18). As a host defense mechanism, pyroptosis controls the release of inflammatory cytokines and danger signals and clears pathogen replication niches [28]. During pyroptotic processes, DNA damage occurs and is associated with reduced DNA laddering and chromatin condensation. As part of the apoptosis process, caspase-activated Dnase (CAD) is used, as well as a restricted CAD inhibitor, to cause DNA damage. However, CAD is not necessary for the process of pyroptosis, during which dimerization activates caspases, inducing the autocleavage and formation of protein complexes with catalytic activity [29,30]. As a result of the cleavage of caspases, some members of the gasdermin gene family are activated, thereby initiating pyroptosis. Generally, as the levels of GSDMDNT processing and GSDMD pores increase, the regulatory mechanism will eventually become disordered, and cell death will occur. This was originally known as pyroptosis, which is a form of necrotic cell death controlled by caspase activation; however, there has been recent evidence that the GSDMDNT domains could also induce pyroptosis without activating caspase; thus, pyroptosis was redefined in 2018 as the programmed death associated with plasma membrane pores that are formed by gasdermin protein family members [18,31].

Gasdermins are evolutionarily conserved cell death effectors; this protein family includes GSDMA, GSDMB, GSDMC, GSDMD, GSDME (DFNA5), and DFNB59. Most members contain two domain structures connected by a flexible linker and exhibit significant similarities between the pore-forming domain and the repressor domain [32]. There is a distinct and restricted pattern of expression for each gasdermin member. An exception is DFNB59, which has a divergent and shorter C-terminus. GSDMD is the protein in the gasdermin family that has been examined in the most detail, and the cleavage site between the two domains is at D276 in mice and D275 in humans, which is mainly cleaved by caspase-1. In addition, it is also possible that other gasdermins can be cleaved by other proteases to induce pyroptosis. Circular RNAs can regulate miR-145, which directly targets GSDMA-mediated pyroptosis in cardiomyocytes. Furthermore, it has been shown that some caspases and granzymes can trigger pyroptosis as a result of proteolytic activation of GSDMB and GSDMC [33,34]. Additionally, a pore-forming domain is present at the amino-terminus of GSDME, which triggers the pyroptosis process, and studies have indicated that Asp270 of GSDME is cleaved by apoptosis-associated caspase-3, resulting in the conversion of apoptotic signals into pyroptotic signals [35]. To date, pyroptosis in DCM is almost entirely GSDMD-mediated. In this article, we review insights into GSDMD in light of the discovery of pyroptosis to provide a direction for future basic research and clinical treatments of DCM.

## 3. GSDMD and the Mechanism of Pore Formation

GSDMD is a member of the gasdermins family, which has now been demonstrated to be capable of forming large pores on the plasma membrane. It functions as an effector molecule involved in pyroptosis downstream of inflammasome signaling pathways and contains a well-defined NT domain of ∼30 kD and a CT domain of ∼26 kD, connected by a flexible linker [36]. Briefly, an autoinhibitory state is maintained by the interaction between the N-terminal and C-terminal domains of GSDMD, where the CT domain of GSDMD has a compact α-helical globular fold, while its NT domain has an extended twisted β-sheet core structure with pore-forming ability. In an inactivated state, the C-terminal of GSDMD inhibits the N-terminal of GSDMD; however, when the pyroptosis process is activated by the cleavages of the two domains of GSDMD, its N-terminal domain with strong affinity for membrane lipids, induces cell lysis by the formation of heterogeneous pores, whose inner diameters range from 10 to 20 nm, and they are sufficiently large to permit the free mobility of miniature proteins, such as mature IL-1β and IL-18 (~4 nm molecular diameter) across the membrane [37]. Due to the fact that the GSDMD pore conduit is predominantly negatively charged, it excludes molecules larger than its diameter as well as acidic molecules. In contrast, the precursors of IL-1β and IL-18 contain an acidic domain that undergoes proteolytic cleavage by caspase-1. This process results in the selective release of mature IL-1β and IL-18 since they exhibit a higher level of basicity than their pro-forms [38]. Definitely, the formation of GSDMD pores plays a crucial role in the execution of pyroptosis, and the inter-domain cleavage of GSDMD serves as a dependable indicator for the activation of inflammatory caspases and the occurrence of cell pyroptosis. The GSDMDNT protein can selectively bind to acidic phospholipids, phosphoinositides, and cardiolipin; however, it also has a weaker affinity for phosphatidic acid and phosphatidylserine to mediate the formation of membrane pores when it oligomerizes [18,39], eventually initiating GSDMD-mediated pyroptosis, leading to the integration of nuclear changes, disrupting ion homeostasis, inducing the swelling and lysis of cells, releasing cytokines, and activating caspases. However, the occurrence of pyroptosis by GSDMD has been demonstrated to confer host protection against bacterial infection [40]. Unlike most pore-forming proteins, when mediating the process of pyroptosis, the GSDMDNT domain can only form membrane pores from the inside of cells, probably because phosphoinositides are present only in the cytoplasmic leaflet of the plasma membrane. This may explain why recombinant proteins containing the GSDMDNT domain could not induce pyroptosis when added directly to cell supernatants [39]. Cardiolipin is a component of the inner mitochondrial membrane. Recent studies have demonstrated that GSDMD could permeabilize through the mitochondrial membrane and form pores within the membrane, eventually releasing cytochrome C and causing pyroptosis [41]. It has also been reported that lipopolysaccharide (LPS), an internalized bacterial endotoxin, participates in pore-forming by activating GSDMD to bind to cardiolipin, which enhances the formation of mitochondrial GSDMD pores and induces the release of mitochondrial DNA (mtDNA) and mitochondrial ROS (mtROS) into the cytosol of endothelial cells, raising mitochondrial dysfunction [42,43]. In recent studies, GSDMD has been shown to be necessary for mtDNA release from neutrophils, and mtDNA acts as a universal DAMP signal, can be oxidized, and promotes GSDMD oligomerization and pore formation, leading to cell death [44]. GSDMD is best known as a pyroptosis executioner. Typically, GSDMD forms pores under different physiological backgrounds, resulting in cell lysis and membrane repair. Gene elimination of GSDMD showed that GSDMD is essential for pyroptosis and the secretion but not proteolytic maturation of IL-1β in both canonical and noncanonical inflammasome responses, in which case pro-caspase-1 is capable of processing GSDMD in an ASC independent way. There has also been evidence that GSDMD pores promote the release of IL-33 by alveolar epithelial MLE-12 cells in vitro [45] and hepatic stellate cells [46] in obesity-associated hepatocellular carcinoma. Recent research has indicated that the process of GSDMD cleavage alone is not sufficient for its pore formation. It is important to note that only GSDMDNT that has been palmitoylated is capable of translocating to the membrane and forming pores [47]. Upon activation of the inflammasome, GSDMD undergoes lipidation by S-palmitoylation at Cys191. Coincidentally, another study also revealed that palmitoylation of GSDMDNT controls GSDMD membrane localization and activation, while GSDMD is palmitoylated at Cys192, in which case a mutation eliminates GSDMD’s ability to localize to plasma membranes [48]. Previous studies have shown that calcium influx through the pores of the GSDMD negatively regulates pyroptosis and acts as an indicator for cells to start membrane repair by recruiting the endosomal sorting complexes required for transport (ESCRT) machinery to damaged membrane regions, such as the plasma membrane. There is no doubt that a complete loss of integrity of the plasma membrane could lead to cell lysis and necrosis, but cells are able to tolerate a limited amount of damage to the plasma membrane, undergo ESCRT-III-mediated repair, and survive [49]. The suppression of ESCRT-III machinery significantly increases the occurrence of pyroptosis and the release of IL-1β in human and murine cells following either canonical or noncanonical inflammasome activation. This ultimately results in the shedding of compromised plasma membranes in the form of exosomes [50]. It is also imperative to note that the ESCRT system only participates in membrane repair to prevent cell lysis without affecting GSDMD activation [50]. Furthermore, Ca^2+^ regulates PMI signaling-mediated cell membrane repair and survival. It was revealed that S660 p-PKCs, which enhanced RelA/Cux transcription factors and the activation of the downstream NF-κB signaling pathway, are able to assess the pore-forming damage through the detection of local Ca^2+^ influx and to promote CXCL1 and CXCL10 expression and secretion [49]. Therefore, the balance between the formation of GSDMD pores and the presence of the membrane repair protein ESCRTIII allows the limited repair of cell membrane damage, which enables the cell to survive in the presence of danger signals. In a recent study, caspase-7 was shown to preserve cell integrity by cleaving and activating acid sphingomyelinase (ASM) to produce ceramide, which promotes endocytic uptake of GSDMD pores and, thus, mediates membrane repair [51]. Interestingly, recent studies have reported that GSDMD pores promote exocytosis by causing Ca-dependent conformational changes that allow intracellular granules to fuse with plasma membranes in mice without inducing pyroptosis [52].

It is believed that GSDMD pores remain in a perpetually open state, and the resulting osmotic imbalance is considered to be extremely harmful. However, a recent study has shown that these pores exhibit phosphoinositide-dependent dynamics, recurrently opening and closing within a period of tens of seconds. Through this circuit, pyroptosis can be tuned pharmacologically, and inflammatory cytokine release by living cells can be controlled [53]. Taken together, these results indicate that the presence of GSDMD pores in the plasma membrane plays an important role in the process of cell pyroptosis. Further investigations are required to fully explain the complete regulation of GSDMD pores and their involvement in the execution of cell pyroptosis.

## 4. Inflammasome-Mediated GSDMD Activation Pathways

**Figure 1:**
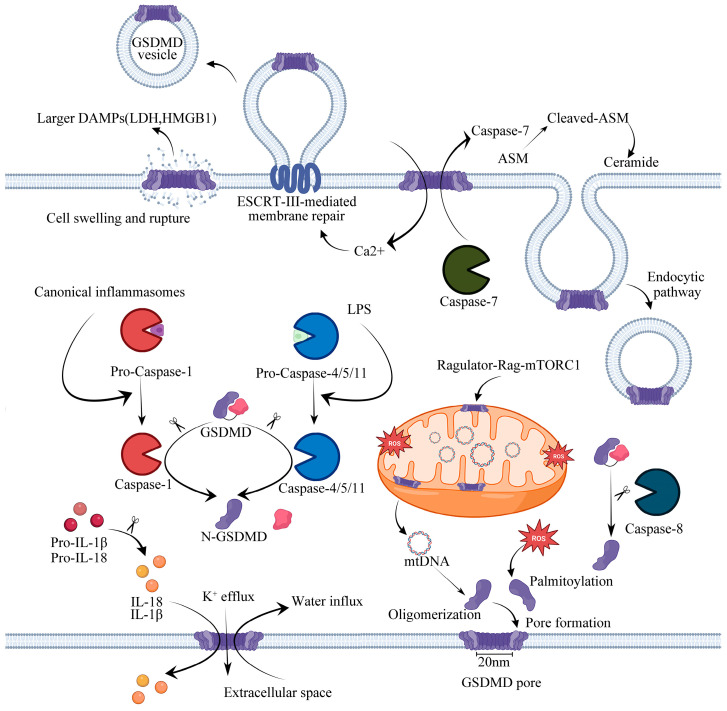
The activation, pore formation, and repair mechanism of GSDMD. The activation of GSDMD is mainly divided into canonical, noncanonical, and other dependent pathways. The canonical pathway depends on caspase-1 via canonical inflammasomes; the noncanonical pathway depends on caspase-4/5/11 via lipopolysaccharide (LPS), while other pathways mainly depend on caspase-8. When stimulated by an external signal, these proteases cleave and activate GSDMD. GSDMDNT can then translocate to the plasma membrane and mitochondria and assemble into transmembrane pores. At the same time, activated forms of IL-1β and IL-18 are released through GSDMD pores to induce pyroptosis. Ca^2+^ influx can trigger the formation of the ESCRT complex and also promote caspase-7 and ASM to meet in the extracellular space, thereby initiating the membrane repair process.

Diabetes mellitus is characterized by a proinflammatory process, whereas inflammation plays a significant role in the development of DCM. DCM is associated with higher expression levels of several cytokines, such as IL-1β, IL-18, and tumor necrosis factor-alpha (TNF-α) [54]. The main pathway leading to pyroptosis involves inflammasomes. Inflammasomes play a significant role in innate immunity, and they are macromolecular complexes that belong to the pattern recognition receptor (PRR) family. Different types of inflammasomes can be triggered by specific pathogen-associated molecular patterns (PAMPs) and damage-associated molecular patterns (DAMPs) in response to exposure to multiple external factors, including hyperglycemia, inflammation, and hyperlipidemia [55]. PRRs identify DAMPs and PAMPs, thereby activating the intrinsic immune reaction. Previous studies have shown that the inflammasome-mediated GSDMD activation pathways that trigger pyroptosis are mainly divided into canonical, noncanonical, and other dependent pathways (Figure 1).

### 4.1. Canonical Pathway

The recognition of PAMPs by PRRs is the critical basis of innate immunity. Cytoplasmic receptor proteins of the PRR, primarily absent in melanoma 2 (AIM2), NOD-like receptor (NLR) family CARD domain-containing protein 4 (NLRC4) and pyrin domain-containing protein 1 (NLRP1), NLRP3, are activated by intracellular PAMPs or DAMPs and assemble to form canonical inflammasomes, recruiting pro-caspase-1, but not always with ASC [56]. The ASC protein is characterized by its bilateral adapter, including the presence of both a PYD domain and a CARD domain, which is dispensable for the formation of some inflammasomes. In the canonical pathway (Figure 1), pro-caspase-1 is cleaved into its biologically active form, named caspase-1, mediating the cleavages of pro-IL-1β and pro-IL-18. Meanwhile, caspase-1 inactivates the autoinhibitory interaction of GSDMD by cleaving the linker between the N-terminal domain and the C-terminal domain of GSDMD. Then, mature forms of IL-1β and IL-18 are released through the membrane via the pores formed by GSDMDNT [36]. Cell swelling and membrane lysis occur when the intracellular and extracellular osmotic pressures are unbalanced due to the presence of too many pores in the cell membrane.

### 4.2. Noncanonical Pathway

A second inflammatory caspase mediates pyroptosis through a noncanonical inflammasome system, which involves the activation of human caspase-4/-5 and homologous murine caspase-11 by intracellular LPS. The caspase-4/-5/-11 protein can be directly bound to and activated by LPS, resulting in pyroptosis [18], whereas caspase-1 is not involved in this pathway (Figure 1). Nevertheless, like caspase-1, caspase-4/5/11 also specifically inactivates the autoinhibitory interaction of GSDMD by cleaving the linker between the GSDMD N-terminal domain and the C-terminal domain, which results in pyroptosis [32]. According to recent studies, the release of neutrophil extracellular traps (NETs) is activated by noncanonical inflammasome signaling (caspase-4/11) and leads to GSDMD-mediated neutrophil death, which can be initiated by extracellularly derived LPS [57]. To date, noncanonical inflammasome-mediated pyroptosis has rarely been reported in DCM. It has only been reported that caspase-11 is not activated in the hearts of diabetic mice but is reduced in the HFD group compared to chow-fed control mice [58]. However, it has been reported that oxidative stress activates caspase-11 to cause GSDMDNT cleavage during myocardial ischemia/reperfusion (I/R) injury, resulting in the oligomerization of GSDMDNT and the formation of membrane pores that mediate pyroptosis [59]. Additionally, in acute kidney injury, caspase-11 mediates GSDMD cleavage and urinary IL-18 excretion. Moreover, caspase-11 promotes the translocation of GSDMD to the plasma membrane, enhancing the formation of pores in primary proximal tubule cells [60]. Therefore, the specific regulatory mechanism of caspase-11 in diabetic cardiomyopathy needs further study. Interestingly, studies have shown that caspase-4/5/11 is different from caspase-1, which cannot directly process IL-1β and IL-18 into its active form, nevertheless promotes GSDMD-mediated potassium efflux, which results in the activation of the noncanonical NLRP3 inflammasome [61,62,63]. This suggests that GSDMD pores result in potassium efflux and play a crucial role in the activation of the inflammatory factor NLRP3.

### 4.3. Other Dependent Pathway

The other dependent inflammasomes include caspase-3 or caspase-8-mediated pyroptosis. Previous studies have shown that chemotherapeutic drugs lead to membrane pore formation and GSDME-mediated pyroptosis through caspase-3 cleavage of GSDME into GSDMENT and GSDMECT [64]. Recent studies have revealed that caspase-8-mediated formation of GSDMD pores is responsible for potassium efflux and activation of the NLRP3 inflammasome [65]. In addition, it is necessary for caspase-8 to dimerize and undergo autoprocessing to promote the cleavage of GSDMD. In summary, these caspases activate and cleave the linker of GSDMD to release autoinhibition of the GSDMDNT domain, resulting in the formation of membrane pores that eventually lead to pyroptosis, which suggests that GSDMD might be a crucial target for future research.

## 5. Molecular Mechanisms of GSDMD-Mediated Pyroptosis in Diabetic Cardiomyopathy

GSDMD-mediated pyroptosis plays a crucial role in the regulation of inflammation, and there could be a close link between GSDMD-mediated pyroptosis and DCM. During the early stages of DCM, the main manifestation is diastolic dysfunction; dysregulated glucose and lipid metabolism lead to increased fatty acid intake and β-oxidation. Over time, increased intracellular fatty acid concentration leads to lipotoxicity, mitochondrial dysfunction, and increased generation of reactive nitrogen species (RNS) and ROS, which together increase endoplasmic reticulum (ER) stress and oxidative stress and inhibit autophagy [66]. These mechanisms result in cardiac hypertrophy, inflammation, and cardiomyocyte death, as well as a progressive profibrotic response that leads to ECM remodeling and fibrosis [67]. These factors ultimately contribute to decreased cardiac contractility and late systolic dysfunction (HFrEF profile) [68]. It has been reported that the protein expression of GSDMDNT was significantly increased in the right atrial appendages of patients with type 2 diabetes mellitus compared with tissues from non-diabetic patients. Moreover, similar results were found in DCM animal models. GSDMDNT was increased both in the left ventricles of C57Bl/6J mice fed with HFD for 8 weeks [58] and mice injected STZ at a dose of 50 mg/kg for 5 consecutive days for 16 weeks, in which GSDMDNT mediated heart inflammation and fibrosis. Moreover, it was reported that GSDMDNT was significantly activated, but full-length GSDMD was reduced in the hearts of 16-week-old male diabetic db/db mice with significant cardiac hypertrophy [69]. In our previous study, GSDMDNT was significantly activated in diabetic mice, which caused significant cardiac systolic and diastolic dysfunction and cardiac hypertrophy [70]. All the above studies revealed the crucial role of GSDMDNT in mediating the pathological process of DCM.

**Figure 2:**
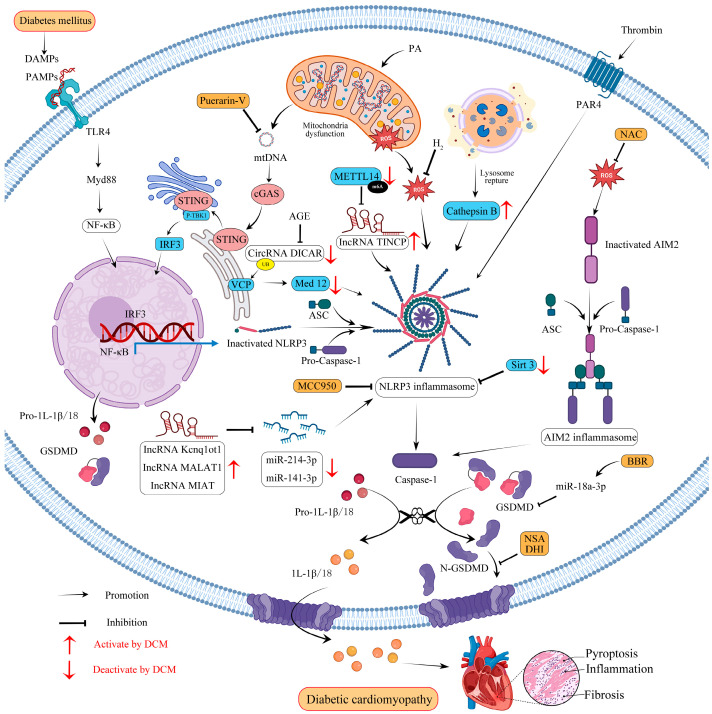
GSDMD-mediated pyroptosis in DCM. Metabolic disorders are crucial to the pathogenesis of DCM. Hyperglycemia and hyperlipidemia affect mitochondrial dysfunction to release mtDNA and mtROS, activate the NFκB signaling pathway, promote the transcription of non-coding RNAs and inflammasome assembly (NLRP3 or AIM2), and activate caspase-1 cleavage of GSDMD to produce an N-terminal GSDMD fragment, which mediates the formation of membrane pores, promoting inflammatory cytokines secretion.

To date, most studies have focused on the canonical pathway, and there is limited research about the noncanonical pathway in DCM. Here, we summarize the pyroptosis pathway in DCM, as shown in Figure 2. Recent studies suggest that the mechanisms regulating GSDMD-mediated pyroptosis in DCM are multifactorial and complex and mainly include ROS, NLRP3, AIM2, mtDNA, and non-coding RNAs.

### 5.1. NLRP3 and ROS Crosstalk

The deleterious effects of diabetes on the heart are increasingly being attributed to inflammatory signals from the NLRP3 inflammasome. NLRP3 recognizes long-range stimuli and responds to a variety of endogenous and infectious DAMPs, including microbial components, pore-forming toxins, nucleic acids, crystals (e.g., cholesterol crystals), and ATP, as well as common cellular stress molecules such as mitochondrial dysfunction, ROS, Ca^2+^, and ruptured lysosomes. With few exceptions, the induction of NLRP3 activity occurs via the stimulation of potassium efflux, leading to the subsequent interaction between NIMA-related kinase 7 (NEK7) and NLRP3 [71,72]. The transcriptional phase of inflammasome priming depends mainly on NF-κB, which regulates the transcription of DNA and the formation of proinflammatory cytokines. NF-κB is activated by pathogens or damage-associated signals, subsequently mediating the transcription of NLRP3 and promoting the activation of the inflammasome. Then, GSDMD pores are formed by the processing of cleaved caspase-1 to release proinflammatory cytokines and DAMPs, finally inducing cell pyroptosis [18]. Under high glucose stimulation, NF-κB is translocated from the cytoplasm to the nucleus in cardiomyocytes, triggering transcriptional activation of NLRP3 [73]. Inhibition of NF-κB/NLRP3 inflammasome-mediated cardiomyocyte pyroptosis has been reported to reduce inflammatory cytokines and improve DCM [74]. In addition, NLRP3 knockout significantly reduced GSDMD and GSDMDNT protein levels in DCM mice [75]. Treatment with MCC950, which is known as an inhibitor of NLRP3, significantly reduced the damaged cardiac function and significantly inhibited the expression of NLRP3 and GSDMDNT in the hearts of DCM rats [76]. Studies have reported that the combination of syringin and tilianin therapy significantly suppressed the up-regulation of TLR4, MyD88, and NF-κB/NLRP3, effectively exerted anti-diabetic effects, and improved cardiac function in diabetic rats [77]. Recently, it was reported that cathepsin B (CTSB), a member of lysosomal cathepsin, can bind directly to NLRP3 and, thus, increase NLRP3/caspase-1/GSDMD signaling pathway-mediated cardiomyocyte pyroptosis in diabetic mice, while NLRP3 knockout mice were able to eliminate the destabilizing effect of CTSB overexpression [75]. To date, increasing research has focused on GSDMD-mediated pyroptosis in the diabetic heart via the canonical NLRP3 inflammasome pathway. For instance, PAR4 genetic deletion has been reported to inhibit HFD-induced caspase-1 and GDSMD cleavage via the canonical NLRP3 inflammasome pathway in diabetic hearts [58]. Furthermore, recent studies have indicated that the administration of exogenous spermidine (SPD) improved cardiac function, decreased collagen deposition and mRNA expression levels of NLRP3, caspase-1, GSDMDNT, IL-1β, IL-17A, and IL-18 in db/db mice, and decreased collagen secretion by reducing GSDMD-mediated pyroptosis in primary neonatal mouse cardiac fibroblasts treated with high glucose [78].

Oxidative stress occurs when there is an imbalance between the production of ROS and their elimination by oxidative defense systems. During DCM progression, both hyperlipidemia and hyperglycemia could induce overproduction of ROS that further promote the oligomerization of NLRP3 as well as inflammatory programmed cell death [75]. ROS is one of the most potent DAMPs to trigger oxidative stress in cells and is a ubiquitous signaling molecule in biological systems. In humans, a deficiency of ROS can also lead to chronic and extreme bacterial infections, which can cause excessive inflammation, leading to pathologies [79]. Recent studies have shown that ROS directly targets GSDMD and increases its activity by oxidatively modifying cysteine 192 (C192) [80]. In addition, ROS is crucial in the formation of GSDMD pores. Studies have shown that ROS generated by LPS is essential for GSDMD palmitoylation, which can be significantly inhibited by reducing ROS [47,48]. Importantly, ROS is closely related to the induction of inflammasomes, particularly NLRP3 in DCM, and inflammasomes act as innate immune signals that trigger pyroptosis. Interestingly, ROS is also involved in inflammation related to the NF-κB and TLR4 pathways in DCM. ROS production in cardiomyocytes is a vicious cycle that leads to further production of ROS, excessive lipid peroxidation, damage to mitochondrial DNA, and activation of inflammation, eventually leading to cell death. Hence, the accumulation of excess lipids impairs respiration as well as mitochondrial biogenesis, which contributes to mitochondrial dysfunction. DCM is associated with the accumulation of ROS, which can induce the activations of a series of downstream signaling pathways. For instance, Exendin-4 has been reported to reduce NLRP3/caspase-1-dependent pyroptosis in diabetic cardiomyocytes by regulating the ROS–AMPK–TXNIP signaling pathway [81], where AMPK activation is driven by oxidative stress through ROS-dependent phosphorylation. Pharmacological ROS inhibitors noticeably reduce NF-κB phosphorylation, the NLRP3 inflammasome, and mature IL-1β in H9c2 cells exposed to high glucose levels; meanwhile, pharmacological NF-κB inhibitors show a similar effect to reduce NLRP3 inflammasome activation, which indicates that ROS and NF-κB may serve as activators of the NLRP3 inflammasome [82]. In addition, mitochondrial ROS accumulation is the main factor leading to mitochondrial dysfunction; the oligomerization and pore formation of GSDMD are controlled by mitochondrial health and ROS production [83]. Recent studies have reported that palmitic acid (PA, 400 μM) impairs mitochondrial function, results in excessive mtROS production, and ultimately leads to GSDMD-dependent cardiomyocyte pyroptosis in DCM [70].

### 5.2. AIM2 and ROS Crosstalk

AIM2 belongs to the AIM2-like receptor family, which has one or two HIN domains and NT PYD [84], and the AIM2 inflammasome specifically binds to and is regulated by cytoplasmic double-stranded DNA (dsDNA) derived from bacteria, viruses, or the host. Once PYD recognizes dsDNA, AIM2 recruits ASC, which further activates caspase-1, resulting in cytokine maturation and release, which plays a role in the regulation of pyroptosis, as well as immune function [84]. It has been reported that AIM2 protein levels are higher in diabetic myocardial tissue, while silencing the AIM2 gene alleviates diabetic type II cardiomyopathy via GSDMDNT-related pyroptosis in diabetic rats. It has also been demonstrated that AIM2 gene silencing reduces GSDMDNT levels in HG-induced H9c2 cardio-myoblasts [85]. In addition, the production of ROS was enhanced by HG treatment in a concentration-dependent manner. After using the ROS inhibitor NAC to inhibit the production of ROS, the protein expression of AIM2 was significantly reduced. Similarly, a recent study reported that the activation of the AIM2 inflammasome was prevented when ROS-induced release of dsDNA from the nucleus was inhibited [86]. Metformin has also been reported to alleviate diabetes-induced macrophage dysfunction by negatively regulating cytoplasmic dsDNA/AIM2, which could activate caspase-1 and cleave GSDMD-mediated pyroptosis [87]. Moreover, elevated levels of ROS within mitochondria can not only induce DNA oxidation and activate the NLRP3 inflammasome [88] but can promote nuclear DNA damage and the activation of AIM2 inflammasomes [89,90], suggesting that mitochondrial function is essential for AIM2/caspase-1/GSDMD pathway-dependent pyroptosis.

### 5.3. mtDNA

Maintaining normal systolic heart function requires abundant energy levels, which is mainly mediated by oxidative phosphorylation in mitochondria [91]. Mitochondria can release multiple DAMPs, which trigger sterile inflammation by activating PRRs that recognize endogenous DAMPs.

Cytoplasmic mtDNA is a DAMP in sterile inflammation following oxidative damage by the accumulation of mtROS [92]. The role of mtDNA in regulating cardiac pyroptosis has been reported in DCM [70,92]. The heart contains a large number of mitochondria for the production of ATP. However, during pathological stress, a large amount of mtDNA could be released from the mitochondria, acting as a potent DAMP and raising the formation of AIM2 [73] or NLRP3 [70] inflammasomes. Recently, it was discovered that mtDNA released from damaged mitochondria mediated cardiac injury in db/db mice via the cGAS–STING–NF-κB signaling pathway [92]. Coincidentally, another article by our team proved that as a result of PA-induced lipotoxicity, mtDNA was released into the cytoplasm and promoted pyroptosis in myocardial cells via the cGAS–STING–GSDMD-mediated pathway [70]. Recently, studies have suggested that GSDMD deficiency reduces hypertrophy and pyroptosis in cardiomyocytes induced by Ang II or TAC in mice, while GSDMD and STING knockout reduce cardiac hypertrophy in mice overexpressing GSDMD in cardiac muscle [93]. Mechanistically, the leakage of mtDNA into the cytoplasm caused by Ang II was suppressed by silencing GSDMD in NRPCs [93]. Similarly, another study also reported that GSDMD was significantly upregulated in the TAC mice and that GSDMDNT was cleaved to induce pyroptosis. Furthermore, it has been claimed that cardiac-specific knockout of GSDMD abolished TAC-induced cardiomyocyte pyroptosis and decreased the release of IL-18 [94]. In addition, studies have shown that cytosolic mtDNA participates in the regulation of odontoblast inflammation and pyroptosis in a STING-dependent manner [95].

It has been proven that GSDMDNT can permeabilize the mitochondrial membrane and induce mitochondrial dysfunction. During these processes, mtDNAs are recognized by the dsDNA sensor cGAS, promoting the generation of cGAMP, which will activate STING and restrain endothelial cell proliferation by downregulating YAP1 signaling [42]. In addition, compared with healthy patients, the increase in serum levels of free mtDNA in type 2 diabetes mellitus (T2DM) patients is positively correlated with pathology, suggesting that GSDMD may form mitochondrial pores to release mtDNA in cardiomyocytes and that mtDNA plays a crucial role in GSDMD-mediated pyroptosis in T2DM.

### 5.4. CircRNAs

Circular RNAs (CircRNAs), a type of non-coding RNA, are characterized by covalently closed loop structures without 3′ tails and 5′ caps and are involved in gene regulation in various pathological and physiological processes, including cardiovascular diseases and T2DM. Current research suggests that circRNAs are the key connection between DCM and pyroptosis. Recent studies have reported that the circular RNA circ_0071269 enhances GSDMA-mediated pyroptosis in cardiomyocytes in DCM by selectively targeting miR-145 [96]. A recent study has shown that circRNA DICAR was suppressed in diabetic mouse hearts, while DCM was improved in DICAR-overexpressing Tg mice [97]. Mechanistically, it has been revealed that DICAR binds directly to valosin-containing protein (VCP), which is expressed in the endoplasmic reticulum (ER), and regulates NLRP3/caspase-1/GSDMD-mediated pyroptosis through VCP-mediated degradation of Med12 by the ubiquitin–proteasome (Ub-Pr) system.

### 5.5. lncRNAs

A long non-coding RNA (LncRNA) is a transcript with a length of more than 200 nucleotides that is considered a non-protein-coding RNA. Numerous research studies have demonstrated that lncRNAs contribute significantly to the pathogenesis of DCM. Kcnq1ot1, which is a lncRNA, inhibits several genes associated with its domain through DNA-modifying proteins, as well as chromatin recruitment. It has been proven that Kcnq1ot1 is upregulated in the diabetic heart, whereas silencing the Kcnq1ot1/miR-214-3p/caspase-1 signaling pathway improves cardiac function and fibrosis, which may partially result from the reduction of GSDMD-mediated pyroptosis in diabetic mice [98]. Interestingly, there is an increase in the IncRNA myocardial infarction-associated transcription (MIAT) allele in patients with DCM, which has also been proven to regulate caspase-1/GSDMD-mediated pyroptosis in vivo and in vitro by targeting miR-214-3p in DCM [99]. Moreover, studies have shown that lncRNA-MALAT1 promotes HG-induced GSDMD-dependent pyroptosis of H9c2 cardiomyocytes by targeting miR-141-3p [100]. Additionally, a recent study reported that pomegranate peel extracts improved DCM through downregulation of lncRNA-MALAT1 and blocking of the NLRP3/caspase-1 signaling pathway [101]. An important epigenetic regulatory factor for lncRNA is N6-methyladenosine (m6A). Recently, a study revealed that METTL14-mediated methylation of m6A and targeting lncRNA-TINCR reduced NLRP3/caspase-1/GSDMD signaling pathway-dependent pyroptosis in DCM [76]. It has been reported that lncRNA is closely related to SIRT3. In cardiovascular diseases, the study revealed that diabetic mice with a deletion of SIRT3 displayed exacerbated cardiac dysfunction, excessive ROS accumulation, increased activation of the NLRP3 inflammasome, and an increased LDH level in the serum [102]. The latest research has indicated that the overexpression of lncRNA-HOTAIR inhibits the HG-induced GSDMD-mediated pyroptosis and inflammatory response in diabetic cardiomyocytes through the FUS/SIRT3 axis [103]. In addition, cardiomyocyte damage caused by hypoxia/reoxygenation (H/R) can be alleviated by the overexpression of HOTAIR or SIRT3, thereby promoting cell viability and decreasing LDH levels [104]. All the above studies suggest that lncRNA plays a crucial role in cardiovascular disease by regulating the FUS/SIRT3 axis.

GSDMD-dependent pyroptosis has been recently characterized in acute kidney injury, myocardial infarction, myocardial ischemia–reperfusion, pathological cardiac hypertrophy, and atherosclerosis, but the role of GSDMD-dependent pyroptosis in diabetes-mediated cardiac remodeling and underlying mechanisms have not been assessed in detail. The signaling pathway of GSDMD-mediated pyroptosis in in vivo and in vitro models of DCM is summarized in Table 1.

## 6. Therapeutic Potential of GSDMD in DCM

Preclinical research has demonstrated the involvement of GSDMD-mediated pyroptosis in the progression of DCM. Here, we summarize the potential therapeutic strategies targeting GSDMD to treat DCM, including clinical drugs, GSDMD inhibitors, herbal medicine, and monomers, to provide a roadmap for the integration of knowledge about GSDMD and DCM.

### 6.1. Clinical Drugs

Accumulating evidence suggests that metformin improves diabetes-related diseases, including DCM, partially by inhibiting GSDMD-mediated pyroptosis. It has been reported that metformin suppressed NLRP3 inflammasome activation and ameliorated diabetes-accelerated atherosclerosis in apoE^−/−^ mice; meanwhile, it blocked the activation of the NLRP3 inflammasome in macrophages induced by HG through the activation of the AMPK pathway in vitro [105]. Moreover, metformin can alleviate inflammation and GSDMD-mediated pyroptosis through the AMPK/mTOR signaling pathway in DCM. However, these effects were reversed by AMPK inhibitors in vivo and in vitro [106]. Nonetheless, the use of metformin is limited because of its side effects when used in large doses. Researchers recently found that hydrogen ameliorated cardiac dysfunction and abnormal morphological structure in STZ-induced diabetic mice by reducing GSDMD-dependent pyroptosis via the AMPK/mTOR signaling pathway. Further studies have also found that hydrogen co-administration with metformin demonstrated potent protective effects as compared to metformin administration alone [107]. These studies support the use of metformin for DCM treatment by inhibiting GSDMD-mediated pyroptosis. However, long-term metformin treatment might cause side effects. Recent studies have demonstrated that long-term metformin treatment activates AMPK and downstream cytochrome C oxidase, leading to GSDME-mediated hepatocyte pyroptosis in HFD-fed Lepr-KO rats [108]. Furthermore, there were similar imitations of caspase-5 and caspase-11 expressions in the study [108], which indicated that metformin-induced pyroptosis of Lepr-deficient hepatocytes involves both canonical and noncanonical pathways.

Ranolazine is effective in the treatment of cardiovascular disease. As a selective cardiac sodium channel inhibitor, it can block late sodium channels to alleviate chronic stable angina pectoris. Moreover, ranolazine improves heart failure by partially restoring Ca^2+^ handling disorders and cellular energy metabolism. There is increasing evidence that ranolazine alleviates diabetes-caused cardiac injury. For instance, studies have demonstrated that ranolazine inhibits apoptosis via activating the NOTCH1/NRG1 signaling pathway in DCM [109]. Meanwhile, it improves cardiac function in diabetic rats by reducing collagen deposition and GSDMD-mediated pyroptosis via upregulating miR-135b [110], implying that ranolazine may have clinical value as a treatment for DCM.

SGLT2 inhibitors are a novel class of hypoglycemic medications that prevent the reabsorption of glucose in the renal proximal tubules, thereby increasing the excretion of glucose through urine. Studies have revealed that SGLT2 inhibition attenuates the activity of NLRP3 inflammasomes in diabetics with cardiovascular disease by inhibiting ketones and insulin [111]. Interestingly, studies have shown that empagliflozin reduces GSDMD-mediated pyroptosis and ameliorates cardiomyopathy via the sGC–cGMP–PKG pathway in db/db mice [69]. A number of clinical studies have demonstrated the effectiveness of empagliflozin in the treatment of type 2 diabetes and heart failure [112,113]. It has been reported that empagliflozin attenuates diabetic pancreatic tissue damage by inhibiting the NLRP3/caspase-1/GSDMD pathway in pancreatic β cells [114]. Recently, it has been reported that empagliflozin effectively suppresses excessive autophagy by regulating the AMPK/GSK3β signaling pathway to improve DCM [115]. This evidence demonstrates the importance of empagliflozin in the clinical treatment of DCM.

### 6.2. GSDMD Inhibitors

Currently, gasdermins are key mediators of pyroptosis, which drives the extension and refinement of tools and approaches to characterize pyroptotic signaling pathways and improve the development of small molecule inhibitors of pyroptosis [91]. Several compounds have been shown to exert inhibitory effects on GSDMD-mediated pyroptosis.

Punchicalagin, an antioxidant polyphenol found in pomegranates, was the first compound shown to block pyroptosis. It was found that punicalagin has remarkable similarities to knocking out GSDMD. The underlying mechanism is that punicalagin may block the plasma membrane fluidity and prevent the correct insertion of GSDMDNT into the plasma membrane, as well as its oligomerization and formation of pores [116]. In 2018, necrosulfonamide (NSA) was found to have specific binding to Cys191/Cys192 of human/mouse GSDMD, which inhibited the formation of membrane pores and IL-1β release in murine monocytes/macrophages. Furthermore, NSA treatment has been shown to inhibit the release of IL-1β in vivo and in vitro [117]. Recently, NSA was chosen as a positive control to inhibit pyroptosis of J774A.1 cells through hydrophobic interaction with mus-GSDMD Cys192 [118]. Moreover, research has revealed that when delivered to cells or as endogenous fumarate, the compound dimethyl fumarate (DMF) undergoes a reaction with GSDMD at critical cysteine residues, resulting in the formation of S-(2-succinyl)-cysteine, which subsequently leads to the succination of GSDMD. The succination of GSDMD decreases its ability to interact with caspases, limiting its ability to be processed and oligomerized and causing pyroptosis [119]. Disulfiram, which is used in the treatment of alcohol addiction, can specifically inhibit GSDMD pore formation. It was found to covalently modify the Cys192 of mouse GSDMD or the Cys191 of human GSDMD to block the oligomerization and pore formation of GSDMD and, ultimately, reduce the release of IL-1β and pyroptosis [120]. Recent studies have shown that a potent component of Danhong injection (DHI), salvianolic acid E (SAE), inhibits the formation of pores in the human GSDMD by binding directly to Cys192 of the mouse GSDMD (homologous to human Cys191) [118]. To date, multiple GSDMD inhibitors have been identified, presenting different perspectives for future treatment of GSDMD-mediated pyroptosis in DCM.

### 6.3. Herbal Medicine and Monomers

Traditional herbal medicine has been used in clinical practice in China for thousands of years and shows great potential in inhibiting inflammation and pyroptosis. It has been shown that herbal medicine has promising efficacy and is even superior to some conventional therapies due to the diverse bioactive phytochemicals and molecular targets, and side effects are rarely observed in clinical practice [121].

Salidroside (SAL) is the primary active ingredient of the Rhodiola species, a traditional herbal medicine used in Europe and Asia for its numerous medicinal properties. Recent studies have reported that SAL alleviates GSDMD-related pyroptosis and ROS generation, while AMPK inhibition abolishes the inhibitory effects of SAL on pyroptosis in HG or HG plus high insulin (HG-HI)-treated INS-1 cells [122]. Hirudin is a natural component found in the salivary glands of blood-sucking leeches, which has been used to promote blood circulation as a traditional Chinese medicine from ancient times in China. Recent studies have found that hirudin alleviates kidney injury and blocks GSDMD-mediated pyroptosis in STZ-induced diabetic nephropathy (DN) mice and further revealed that hirudin inhibits GSDMD expression via regulating Irf2 [123]. Salvianolic acid A (SAA) is the main biologically active ingredient of Danshen, which is widely used as a Traditional Chinese Medicine, with a variety of drug properties, especially in the prevention and treatment of cardiovascular and metabolic diseases. It was demonstrated that SAA reduced pyroptosis in endothelial cells of the aortic sinus in Western diet-fed diabetic ApoE^−/−^ mice, and reduced GSDMD-dependent pyroptosis by downregulating the PKM2/PKR pathway in HG-treated endothelial cells in vitro [124].

To date, several herbal medicines specifically targeting Gasdermin D have been illustrated. Danhong injection (DHI) was the first Chinese herbal medicine to show specific inhibition of GSDMD activity. Studies have demonstrated that DHI can reduce the area of fibrotic tissue in type 2 diabetic mice with myocardial infarction, mainly because it can directly inhibit the oligomerization and formation of GSDMDNT pores in a dose-dependent manner. Specifically, the dose of 25 μL/mL of DHI exhibited more significant suppression of cell pyroptosis compared to a normal dosage of NSA [118]. Puerarin V is a newly identified form of puerarin that has been shown to ameliorate myocardial injury in DCM rats by regulating NLRP3/caspase-1/GSDMD-dependent pyroptosis, and it was further reported that puerarin-V exhibits a greater effect than puerarin, puerarin injections, or metformin in DCM [125]. Gypenosides, which are the main components of *Gynostemma pentaphyllum*, can inhibit ROS-induced activation of the NLRP3 inflammasome caused by cytochrome c, improve myocardial cell injury in H9c2 cells, and inhibit the development of DCM in vivo [126]. Berberine (BBR), which is an extract of a Chinese herb, is a natural compound that exhibits numerous beneficial properties. Recent studies have revealed that BBR significantly improved DCM by inhibiting IL-1β secretion and GSDMD expression at the post-transcriptional level by activating its promoter (−1000/−500) to upregulate miR-18a-3p expression [127]. As a triterpenoid alkaloid, cycloxanthine D (CVB-D) is an extract of Caulis et Ramulus Buxi Sinicae, which is used primarily in Chinese medicine to prevent and treat cardiovascular diseases such as arrhythmias and heart failure. It has been reported that CVB-D improves DCM via inhibition of cardiomyocyte pyroptosis through the NLRP3/caspase-1/GSDMD pathway and reduces HG-induced pyroptosis in PNRCMs [128].

The current findings presented here suggest that herbal medicines and monomers can be used to develop new drugs to target GSDMD-mediated pyroptosis in DCM. The therapeutic methods and mechanisms of GSDMD-mediated pyroptosis in DCM are summarized in Table 2.

## 7. Summary and Discussion

Heart failure can occur as a consequence of DCM, which is a chronic multifactorial complication of T2DM. Pyroptosis is primarily mediated by GSDMD, which is an innate immune effector mechanism and is widely recognized as an inflammatory process that causes cell death. Clinical and preclinical studies have demonstrated that myocardial inflammation plays a significant role in the development of cardiac dysfunction induced by diabetes. Myocardial infarction causes blood leukocytosis, which is correlated negatively with patient survival. Loss of GSDMD caused decreased IL-1β release from neutrophils and, subsequently, reduced neutrophils and monocytes in the infarcted heart [129]. Monocyte macrophages are important immune cells in the body. Studies have shown that diabetes initiates macrophage pyroptosis. Under high blood glucose conditions, macrophage function, such as inflammatory cytokine secretion, phagocytosis, chemotaxis, and immune response, is disturbed. In STZ-induced diabetic cardiomyopathy, there was a significant rise in the formation of inflammasomes, pyroptosis markers, MMP9, and the infiltration of monocytes (CD14), macrophages (iNOS), and dendritic cells (CD11b and CD11c). Studies have shown that the impact of deficiency in CD74, the cognate receptor for the regulatory cytokine macrophage migration inhibitory factor (MIF), improves T2D-induced cardiac remodeling and pyroptosis. In addition, puerarin improves diabetic cardiomyopathy by inhibiting NLRP3-caspase-1-GSDMD-mediated pyroptosis in RAW264.7 macrophage cells [130]. The above studies have demonstrated the role of GSDMD-mediated pyroptosis in promoting diabetes-induced cardiovascular disease. Whether GSDMD-mediated immune cell pyroptosis can be a promising target for DCM treatment seems to be a question worth exploring in the future. More and more studies have shown that the GSDMD-mediated pyroptosis pathway is a participant in the process of cell death with the development of DCM. Furthermore, it has been observed that the mitigation of myocardial pyroptosis is closely associated with the preservation of cardiac function. Many studies have shown that the initiation of pyroptosis may arise from the activation of the NLRP3 inflammasome. However, these processes have a common endpoint, which is the cleavage of GSDMD to form membrane pores and mediate pyroptosis, suggesting that GSDMD could be a putative biomarker of pyroptosis.

In this review, we discussed mechanisms involved in the GSDMD/caspase-1-dependent classical pyroptotic pathway, the GSDMD/caspase-4/5/11-dependent non-classical pyroptotic pathway, and other dependent pathways, as well as the different signaling pathways of GSDMD-mediated pyroptosis in in vivo and in vitro models of DCM. We also highlighted the therapeutic potential of GSDMD in DCM, including the use of clinical drugs, GSDMD inhibitors, and herbal medicine and monomers to inhibit the pyroptotic pathway for therapeutic effects. A series of significant papers have established an essential role for GSDMD-mediated pyroptosis in DCM. Nevertheless, many questions still remain unanswered, and there is no doubt that these studies are only revealing the tip of the iceberg in terms of what is going on. The clinical diagnosis of DCM, especially in the early asymptomatic stage, remains challenging due to the lack of clinical biomarkers with high specificity and sensitivity. Recently, studies have shown that cardiac-specific knockout of GSDMD can improve cardiac remodeling induced by pressure overload and represents a potential biomarker for the diagnosis of cardiac remodeling [94]. Furthermore, overexpression of miR-18a-3p inhibits the expression of GSDMD and improves biomarkers of cardiac function in DCM rats [127], suggesting that GSDMD may be a biomarker for the diagnosis of early clinical DCM. Therefore, the role of GSDMD in DCM in the next few years needs to be further studied.

## Figures and Tables

**Table 1:** The signaling pathway of GSDMD-mediated pyroptosis in in vivo and in vitro models of DCM.

Signaling Pathway	Conditions	Treatments	Model	Pyroptotic Marker				Inflammatory Marker/ROS	Other Cell Death Markers/Relevant Findings	Ref
				GSDMD NT	GSDMD	caspase-1	Others			
PAR4/NLRP3	HFD for 8 weeks	PAR4^−/−^	C57BL/6J mice	↓		↓		IL-1β ↓		[58]
	HFD for 8 weeks	Control	C57BL/6J mice	↑	-	↑	ASC- NLRP3-	IL-1β and IL-18 ↑	P10, p20, p37 caspase-1 ↑	
	Patients with type 2 diabetes mellitus		Atria of patients with type 2 diabetes	↑	-	↑		IL-1β ↑		
CTSB/NLRP3	HG (33.3 mM)	Si-CTSB	NRCMs	↓	↓	↓	NLRP3 ASC ↓		TUNEL, LDH ↓	[75]
	STZ (50 mg/kg) for 5 days	CTSB^−/+^ heterozygous	C57BL/6J mice	↓	↓	↓	NLRP3 ASC ↓	IL-1β and IL-18 ↓	EF, FS ↑ LVEDd, LVESd, TUNEL, CD45, CD68 ↓	
METTL14/TINCR	HG (50 mM) for 24 h	MCC950 (5 μg/mL) for 24 h	H9c2 and NRVMs	↓		↓	NLRP3 ↓	IL-1β and IL-18 ↓		[76]
	HG (50 mM) for 24 h	Overexpression METTL14 + overexpression TINCR	H9c2 and NRVMs	↑		↑	NLRP3 ↑	IL-1β and IL-18 ↑	TUNEL ↓	
	STZ (65 mg/kg)	METTL14 overexpression	SD rats	↓		↓	NLRP3 ↓			
	STZ (65 mg/kg)	MCC950 (10 mg/kg)	SD rats	↓			NLRP3 ↓		EF, FS ↑ CKMB, LDH, and AST ↓	
cGAS-STING/p-TBK1/p-IRF3	STZ (50 mg/kg) for 5 days with HFD	STING-shRNA	Male C57BL/6J mice	↓	-	↓	NLRP3 ↓	TNF-α, IFN-β, IL-1β and IL-18 ↓	E/A, EF, FS ↑WGA, LDH, ANP, BNP, and β-MHC ↓	[70]
	PA (400 μM) for 24 h	PA + EtBr	H9c2 and NMCMs	↓	-	↓	NLRP3 ↓	TNF-α, IFN-β, IL-1β, and IL-18 ↓	mtDNA, LDH, TUNEL, α-actinin, ANP, BNP, and β-MHC ↓	
AIM2	HG 25 (mM) for 24 h	5 mmol/L NAC	H9c2	↓		↓	ASC ↓	ROS ↓	EthD-III, TUNEL ↓	[85]
	HFD for 4 weeks + STZ (40 mg/kg)	AIM2-shRNA	SD rats	↓		↓	ASC ↓	IL-1β ↓	E/A, EF, FS ↑ TUNEL, collagen I, and collagen III ↓	
CircRNA DICAR/VCP/Med12	AGEs (200 μg/mL) for 48 h	Overexpressed DICAR	HL-1 cardiomyocytes		↓	↓	ASC NLRP3 ↓			[97]
	db/db mice	DICAR ^Tg^	C57BL/KsJ mice		↓	↓	ASC NLRP3 ↓		EF, FS ↑ WGA, LVEDd, LVEDV ↓	
		DICAR^+/−^	C57BL/KsJ mice		↑	↑	ASC NLRP3 ↑		EF, FS ↓ LVEDD, LVEDV, WGA ↑	
lncRNA Kcnq1ot1/miR-214-3p	STZ (50 mg/kg) for 5 days	Kcnq1ot1-shRNA	C57BL/6 mice	↓		↓	NLRP3 ↓	IL-1β ↓	EF, FS ↑ TGF-β1, p-smad2, p-smad3, collagen I, and collagen III ↓	[98]
	HG (30 mM) for 24 h	Si-Kcnq1ot1	Cardiac fibroblasts	↓		↓	NLRP3 ↓	IL-1β ↓	TGF-β1, p-smad2, p-smad3, collagen I, and collagen III ↓	
lncRNA MIAT/miR-214-3p	HG (25 mM) for 24 h	Si-MIAT	Cardiac fibroblasts		↓	↓		IL-1β and IL-18 ↓		[99]
	STZ (150 mg/kg)	LV-siMIAT	C57BL/6 mice		↓	↓		IL-1β and IL-18 ↓	EF, FS ↑	
lncRNA MALAT1/miR-141-3p	HG (30 mM) for 24 h	Control	H9C2	↑	↓	↑	ASC ↑ NLRP3 ↑		TUNEL ↑	[100]
		Overexpression MALAT1		↑↑	↓↓	↑↑	ASC ↑↑ NLRP3 ↑↑		TUNEL ↑↑	
		Si-MALAT1		↓	↑	↓	ASC ↓ NLRP3 ↓		TUNEL ↓	
		Overexpression MALAT1 + overexpression-miR-141-3p		↓	↑	↓	ASC ↓ NLRP3 ↓		TUNEL ↓	
lncRNA HOTAIR/FUS/SIRT3	HG (30 mM) for 24 h	Overexpression HOTAIR	H9C2	↓		↓	NLRP3 ↓	IL-1β and IL-18 ↓		[103]
	HG (30 mM) for 24 h	Overexpression SIRT3		↓		↓	NLRP3 ↓	IL-1β and IL-18 ↓		
	HG (30 mM) for 24 h	Overexpression HOTAIR + sh- SIRT3		↑		↑	NLRP3 ↑	IL-1β and IL-18 ↑		

**Table 2:** Therapeutic methods and mechanisms of action on GSDMD-mediated pyroptosis.

Drug Therapies	Dosage	Conditions	Model	Pyroptotic Marker				Inflammatory Marker/ROS	Other Cell Death Markers/Relevant Findings	Ref.
				GSDMDNT	GSDMD	Caspase-1	Others			
Metformin	2 mM metformin	HG (30 mM) for 24 h	Primary cardiomyocytes	↓		↓	NLRP3 ↓	IL-1β ↓		[106]
Ranolazine	30 mg/kg for 12 weeks	8 weeks of HFD + STZ (35 mg/kg)	SD rats		↓	↓	-	IL-1β ↓	EF, FS ↑ TGF-β1, collagen I and collagen III ↓	[110]
Empagliflozin	10 mg/kg/day for 8 weeks		db/db mice	↓	↑	↓	NLRP3 ↓	IL-1β ↓	TUNEL, caspase-3, Bax, SOD2, NOX2/4, ANP, BNP, β-MHC, TGF-β, collagen I and collagen III ↓	[69]
H2	Inhaled 2% hydrogen for 3 h per day	STZ (50 mg/kg) for 5 days	C57BL/6 mice	↓	↑	↓	NLRP3 ↓	IL-18, IL-1β ↓	TGF-β1, p-smad3, p-smad2, COL-I, COL-III, α-SMA ↓	[107]
Puerarin-V	50, 100, or 150 mg/kg/day for 6 weeks	HFD for 6 weeks + STZ (30 mg/kg) + ISO (5 mg/kg)	SD rats	↓		↓	P2X7R, NLRP3,ASC ↓	IL-18, IL-1β ↓	cTn-T, NT-proBNP, AST, LDH, CK-MB, MDA ↓SOD, GSH ↑	[125]
Berberine	200 mg/kg/day for 4 weeks	HFD for 4 weeks + STZ (30 mg/kg) for 5 days	SD rats	↓	↓	-	NLRP3-	IL-1β ↓	cTn-I, CK-MB ↓	[127]
	MiR-18a-3p overexpres- sion	HFD for 4 weeks + STZ (30 mg/kg) for 5 days	SD rats	↓	↓	-	NLRP3-	IL-1β ↓		
	MiR-18a-3p mimics	HG (33 mM) for 2 days	H9C2 cells	↓	↓			IL-1β ↓	PI ↓	
Cyclovirobuxine D	0.5 mg/kg/day or 1 mg/kg/day for 2 months	HFD + STZ (30 mg/kg) for 4 days	C57BL/6 mice	↓	↓	↓	NLRP3 ↓	IL-18, IL-1β ↓	TUNEL, LDH ↓EF, FS ↑	[128]

## References

[1] Cornier M.-A., Dabelea D., Hernandez T.L., Lindstrom R.C., Steig A.J., Stob N.R., Van Pelt R.E., Wang H., Eckel R.H. (2008). The metabolic syndrome. Endocr. Rev..

[2] Jia G., Hill M.A., Sowers J.R. (2018). Diabetic Cardiomyopathy: An Update of Mechanisms Contributing to This Clinical Entity. Circ. Res..

[3] Dillmann W.H. (2019). Diabetic Cardiomyopathy. Circ. Res..

[4] Frustaci A., Kajstura J., Chimenti C., Jakoniuk I., Leri A., Maseri A., Nadal-Ginard B., Anversa P. (2000). Myocardial cell death in human diabetes. Circ. Res..

[5] Wei J., Zhao Y., Liang H., Du W., Wang L. (2022). Preliminary evidence for the presence of multiple forms of cell death in diabetes cardiomyopathy. Acta Pharm. Sin. B.

[6] Strowig T., Henao-Mejia J., Elinav E., Flavell R. (2012). Inflammasomes in health and disease. Nature.

[7] Soengas M.S., Alarcón R.M., Yoshida H., Giaccia A.J., Hakem R., Mak T.W., Lowe S.W. (1999). Apaf-1 and caspase-9 in p53-dependent apoptosis and tumor inhibition. Science.

[8] Dixon S.J., Lemberg K.M., Lamprecht M.R., Skouta R., Zaitsev E.M., Gleason C.E., Patel D.N., Bauer A.J., Cantley A.M., Yang W.S. (2012). Ferroptosis: An iron-dependent form of nonapoptotic cell death. Cell.

[9] Levine B., Mizushima N., Virgin H.W. (2011). Autophagy in immunity and inflammation. Nature.

[10] Degterev A., Huang Z., Boyce M., Li Y., Jagtap P., Mizushima N., Cuny G.D., Mitchison T.J., Moskowitz M.A., Yuan J. (2005). Chemical inhibitor of nonapoptotic cell death with therapeutic potential for ischemic brain injury. Nat. Chem. Biol..

[11] Tsvetkov P., Coy S., Petrova B., Dreishpoon M., Verma A., Abdusamad M., Rossen J., Joesch-Cohen L., Humeidi R., Spangler R.D. (2022). Copper induces cell death by targeting lipoylated TCA cycle proteins. Science.

[12] Liu R., Duan T., Yu L., Tang Y., Liu S., Wang C., Fang W.-J. (2023). Acid sphingomyelinase promotes diabetic cardiomyopathy via NADPH oxidase 4 mediated apoptosis. Cardiovasc. Diabetol..

[13] Kuethe F., Sigusch H.H., Bornstein S.R., Hilbig K., Kamvissi V., Figulla H.R. (2007). Apoptosis in patients with dilated cardiomyopathy and diabetes: A feature of diabetic cardiomyopathy?. Horm. Metab. Res..

[14] Li X., Li Z., Dong X., Wu Y., Li B., Kuang B., Chen G., Zhang L. (2023). Astragaloside IV attenuates myocardial dysfunction in diabetic cardiomyopathy rats through downregulation of CD36-mediated ferroptosis. Phytother. Res..

[15] Xue F., Cheng J., Liu Y., Cheng C., Zhang M., Sui W., Chen W., Hao P., Zhang Y., Zhang C. (2022). Cardiomyocyte-specific knockout of ADAM17 ameliorates left ventricular remodeling and function in diabetic cardiomyopathy of mice. Signal Transduct. Target. Ther..

[16] Zychlinsky A., Prevost M.C., Sansonetti P.J. (1992). Shigella flexneri induces apoptosis in infected macrophages. Nature.

[17] Cookson B.T., Brennan M.A. (2001). Pro-inflammatory programmed cell death. Trends Microbiol..

[18] Broz P., Pelegrín P., Shao F. (2020). The gasdermins, a protein family executing cell death and inflammation. Nat. Rev. Immunol..

[19] Zhang Y., Huang Z., Li H. (2017). Insights into innate immune signalling in controlling cardiac remodelling. Cardiovasc. Res..

[20] Malireddi R.K.S., Kesavardhana S., Kanneganti T.-D. (2019). ZBP1 and TAK1: Master Regulators of NLRP3 Inflammasome/Pyroptosis, Apoptosis, and Necroptosis (PAN-optosis). Front. Cell. Infect. Microbiol..

[21] Hao Y., Yang B., Yang J., Shi X., Yang X., Zhang D., Zhao D., Yan W., Chen L., Zheng H. (2022). ZBP1: A Powerful Innate Immune Sensor and Double-Edged Sword in Host Immunity. Int. J. Mol. Sci..

[22] Kesavardhana S., Malireddi R.K.S., Burton A.R., Porter S.N., Vogel P., Pruett-Miller S.M., Kanneganti T.-D. (2020). The Zα2 domain of ZBP1 is a molecular switch regulating influenza-induced PANoptosis and perinatal lethality during development. J. Biol. Chem..

[23] Zheng M., Kanneganti T.-D. (2020). The regulation of the ZBP1-NLRP3 inflammasome and its implications in pyroptosis, apoptosis, and necroptosis (PANoptosis). Immunol. Rev..

[24] Gitlin A.D., Heger K., Schubert A.F., Reja R., Yan D., Pham V.C., Suto E., Zhang J., Kwon Y.C., Freund E.C. (2020). Integration of innate immune signalling by caspase-8 cleavage of N4BP1. Nature.

[25] Fritsch M., Günther S.D., Schwarzer R., Albert M.-C., Schorn F., Werthenbach J.P., Schiffmann L.M., Stair N., Stocks H., Seeger J.M. (2019). Caspase-8 is the molecular switch for apoptosis, necroptosis and pyroptosis. Nature.

[26] Bertheloot D., Latz E., Franklin B.S. (2021). Necroptosis, pyroptosis and apoptosis: An intricate game of cell death. Cell. Mol. Immunol..

[27] Galluzzi L., Vitale I., Aaronson S.A., Abrams J.M., Adam D., Agostinis P., Alnemri E.S., Altucci L., Amelio I., Andrews D.W. (2018). Molecular mechanisms of cell death: Recommendations of the Nomenclature Committee on Cell Death 2018. Cell Death Differ..

[28] Orning P., Lien E., Fitzgerald K.A. (2019). Gasdermins and their role in immunity and inflammation. J. Exp. Med..

[29] Jorgensen I., Miao E.A. (2015). Pyroptotic cell death defends against intracellular pathogens. Immunol. Rev..

[30] Lee B.L., Stowe I.B., Gupta A., Kornfeld O.S., Roose-Girma M., Anderson K., Warming S., Zhang J., Lee W.P., Kayagaki N. (2018). Caspase-11 auto-proteolysis is crucial for noncanonical inflammasome activation. J. Exp. Med..

[31] Shao R., Lou X., Xue J., Ning D., Chen G., Jiang L. (2022). Review: The role of GSDMD in sepsis. Inflamm. Res..

[32] Shi J., Zhao Y., Wang K., Shi X., Wang Y., Huang H., Zhuang Y., Cai T., Wang F., Shao F. (2015). Cleavage of GSDMD by inflammatory caspases determines pyroptotic cell death. Nature.

[33] Zhou Z., He H., Wang K., Shi X., Wang Y., Su Y., Wang Y., Li D., Liu W., Zhang Y. (2020). Granzyme A from cytotoxic lymphocytes cleaves GSDMB to trigger pyroptosis in target cells. Science.

[34] Hou J., Zhao R., Xia W., Chang C.-W., You Y., Hsu J.-M., Nie L., Chen Y., Wang Y.-C., Liu C. (2020). PD-L1-mediated gasdermin C expression switches apoptosis to pyroptosis in cancer cells and facilitates tumour necrosis. Nat. Cell Biol..

[35] Rogers C., Fernandes-Alnemri T., Mayes L., Alnemri D., Cingolani G., Alnemri E.S. (2017). Cleavage of DFNA5 by caspase-3 during apoptosis mediates progression to secondary necrotic/pyroptotic cell death. Nat. Commun..

[36] Devant P., Kagan J.C. (2023). Molecular mechanisms of gasdermin D pore-forming activity. Nat. Immunol..

[37] Dai Z., Liu W.-C., Chen X.-Y., Wang X., Li J.-L., Zhang X. (2023). Gasdermin D-mediated pyroptosis: Mechanisms, diseases, and inhibitors. Front. Immunol..

[38] Xia S., Zhang Z., Magupalli V.G., Pablo J.L., Dong Y., Vora S.M., Wang L., Fu T.-M., Jacobson M.P., Greka A. (2021). Gasdermin D pore structure reveals preferential release of mature interleukin-1. Nature.

[39] Ding J., Wang K., Liu W., She Y., Sun Q., Shi J., Sun H., Wang D.-C., Shao F. (2016). Erratum: Pore-forming activity and structural autoinhibition of the gasdermin family. Nature.

[40] Wang J., Deobald K., Re F. (2019). Gasdermin D Protects from Melioidosis through Pyroptosis and Direct Killing of Bacteria. J. Immunol..

[41] Rogers C., Alnemri E.S. (2019). Gasdermins: Novel mitochondrial pore-forming proteins. Mol. Cell Oncol..

[42] Huang L.S., Hong Z., Wu W., Xiong S., Zhong M., Gao X., Rehman J., Malik A.B. (2020). mtDNA Activates cGAS Signaling and Suppresses the YAP-Mediated Endothelial Cell Proliferation Program to Promote Inflammatory Injury. Immunity.

[43] Weindel C.G., Martinez E.L., Zhao X., Mabry C.J., Bell S.L., Vail K.J., Coleman A.K., VanPortfliet J.J., Zhao B., Wagner A.R. (2022). Mitochondrial ROS promotes susceptibility to infection via gasdermin D-mediated necroptosis. Cell.

[44] Miao N., Wang Z., Wang Q., Xie H., Yang N., Wang Y., Wang J., Kang H., Bai W., Wang Y. (2023). Oxidized mitochondrial DNA induces gasdermin D oligomerization in systemic lupus erythematosus. Nat. Commun..

[45] Chen W., Chen S., Yan C., Zhang Y., Zhang R., Chen M., Zhong S., Fan W., Zhu S., Zhang D. (2022). Allergen protease-activated stress granule assembly and gasdermin D fragmentation control interleukin-33 secretion. Nat. Immunol..

[46] Yamagishi R., Kamachi F., Nakamura M., Yamazaki S., Kamiya T., Takasugi M., Cheng Y., Nonaka Y., Yukawa-Muto Y., Thuy L.T.T. (2022). Gasdermin D-mediated release of IL-33 from senescent hepatic stellate cells promotes obesity-associated hepatocellular carcinoma. Sci. Immunol..

[47] Du G., Healy L.B., David L., Walker C., Fontana P., Dong Y., Devant P., Puthenveetil R., Ficarro S.B., Banerjee A. (2023). ROS-dependent palmitoylation is an obligate licensing modification for GSDMD pore formation. bioRxiv.

[48] Balasubramanian A., Ghimire L., Hsu A.Y., Kambara H., Liu X., Hasegawa T., Xu R., Tahir M., Yu H., Lieberman J. (2023). Palmitoylation of gasdermin D directs its membrane translocation and pore formation in pyroptosis. bioRxiv.

[49] Wang W., Prokopec J.S., Zhang Y., Sukhoplyasova M., Shinglot H., Wang M.-T., Linkermann A., Stewart-Ornstein J., Gong Y.-N. (2022). Sensing plasma membrane pore formation induces chemokine production in survivors of regulated necrosis. Dev. Cell.

[50] Rühl S., Shkarina K., Demarco B., Heilig R., Santos J.C., Broz P. (2018). ESCRT-dependent membrane repair negatively regulates pyroptosis downstream of GSDMD activation. Science.

[51] Nozaki K., Maltez V.I., Rayamajhi M., Tubbs A.L., Mitchell J.E., Lacey C.A., Harvest C.K., Li L., Nash W.T., Larson H.N. (2022). Caspase-7 activates ASM to repair gasdermin and perforin pores. Nature.

[52] Zhang J., Yu Q., Jiang D., Yu K., Yu W., Chi Z., Chen S., Li M., Yang D., Wang Z. (2022). Epithelial Gasdermin D shapes the host-microbial interface by driving mucus layer formation. Sci. Immunol..

[53] Santa Cruz Garcia A.B., Schnur K.P., Malik A.B., Mo G.C.H. (2022). Gasdermin D pores are dynamically regulated by local phosphoinositide circuitry. Nat. Commun..

[54] Bugger H., Abel E.D. (2014). Molecular mechanisms of diabetic cardiomyopathy. Diabetologia.

[55] Feng S., Fox D., Man S.M. (2018). Mechanisms of Gasdermin Family Members in Inflammasome Signaling and Cell Death. J. Mol. Biol..

[56] Xia S., Hollingsworth L.R., Wu H. (2020). Mechanism and Regulation of Gasdermin-Mediated Cell Death. Cold Spring Harb. Perspect. Biol..

[57] Chen K.W., Monteleone M., Boucher D., Sollberger G., Ramnath D., Condon N.D., von Pein J.B., Broz P., Sweet M.J., Schroder K. (2018). Noncanonical inflammasome signaling elicits gasdermin D-dependent neutrophil extracellular traps. Sci. Immunol..

[58] Fender A.C., Kleeschulte S., Stolte S., Leineweber K., Kamler M., Bode J., Li N., Dobrev D. (2020). Thrombin receptor PAR4 drives canonical NLRP3 inflammasome signaling in the heart. Basic Res. Cardiol..

[59] Shi H., Gao Y., Dong Z., Yang J.e., Gao R., Li X., Zhang S., Ma L., Sun X., Wang Z. (2021). GSDMD-Mediated Cardiomyocyte Pyroptosis Promotes Myocardial I/R Injury. Circ. Res..

[60] Miao N., Yin F., Xie H., Wang Y., Xu Y., Shen Y., Xu D., Yin J., Wang B., Zhou Z. (2019). The cleavage of gasdermin D by caspase-11 promotes tubular epithelial cell pyroptosis and urinary IL-18 excretion in acute kidney injury. Kidney Int..

[61] Baker P.J., Boucher D., Bierschenk D., Tebartz C., Whitney P.G., D’Silva D.B., Tanzer M.C., Monteleone M., Robertson A.A.B., Cooper M.A. (2015). NLRP3 inflammasome activation downstream of cytoplasmic LPS recognition by both caspase-4 and caspase-5. Eur. J. Immunol..

[62] Rühl S., Broz P. (2015). Caspase-11 activates a canonical NLRP3 inflammasome by promoting K^+^ efflux. Eur. J. Immunol..

[63] Linder A., Hornung V. (2020). Irgm2 and Gate-16 put a break on caspase-11 activation. EMBO Rep..

[64] Wang Y., Gao W., Shi X., Ding J., Liu W., He H., Wang K., Shao F. (2017). Chemotherapy drugs induce pyroptosis through caspase-3 cleavage of a gasdermin. Nature.

[65] Orning P., Weng D., Starheim K., Ratner D., Best Z., Lee B., Brooks A., Xia S., Wu H., Kelliher M.A. (2018). Pathogen blockade of TAK1 triggers caspase-8-dependent cleavage of gasdermin D and cell death. Science.

[66] Kenny H.C., Abel E.D. (2019). Heart Failure in Type 2 Diabetes Mellitus. Circ. Res..

[67] Hölscher M.E., Bode C., Bugger H. (2016). Diabetic Cardiomyopathy: Does the Type of Diabetes Matter?. Int. J. Mol. Sci..

[68] van Heerebeek L., Hamdani N., Handoko M.L., Falcao-Pires I., Musters R.J., Kupreishvili K., Ijsselmuiden A.J.J., Schalkwijk C.G., Bronzwaer J.G.F., Diamant M. (2008). Diastolic stiffness of the failing diabetic heart: Importance of fibrosis, advanced glycation end products, and myocyte resting tension. Circulation.

[69] Xue M., Li T., Wang Y., Chang Y., Cheng Y., Lu Y., Liu X., Xu L., Li X., Yu X. (2019). Empagliflozin prevents cardiomyopathy via sGC-cGMP-PKG pathway in type 2 diabetes mice. Clin. Sci..

[70] Yan M., Li Y., Luo Q., Zeng W., Shao X., Li L., Wang Q., Wang D., Zhang Y., Diao H. (2022). Mitochondrial damage and activation of the cytosolic DNA sensor cGAS-STING pathway lead to cardiac pyroptosis and hypertrophy in diabetic cardiomyopathy mice. Cell Death Discov..

[71] He Y., Zeng M.Y., Yang D., Motro B., Núñez G. (2016). NEK7 is an essential mediator of NLRP3 activation downstream of potassium efflux. Nature.

[72] Muñoz-Planillo R., Kuffa P., Martínez-Colón G., Smith B.L., Rajendiran T.M., Núñez G. (2013). K⁺ efflux is the common trigger of NLRP3 inflammasome activation by bacterial toxins and particulate matter. Immunity.

[73] Lu Y., Lu Y., Meng J., Wang Z. (2021). Pyroptosis and Its Regulation in Diabetic Cardiomyopathy. Front. Physiol..

[74] Qu X.-F., Zhai B.-Z., Hu W.-L., Lou M.-H., Chen Y.-H., Liu Y.-F., Chen J.-G., Mei S., You Z.-Q., Liu Z. (2022). Pyrroloquinoline quinone ameliorates diabetic cardiomyopathy by inhibiting the pyroptosis signaling pathway in C57BL/6 mice and AC16 cells. Eur. J. Nutr..

[75] Liu C., Yao Q., Hu T., Cai Z., Xie Q., Zhao J., Yuan Y., Ni J., Wu Q.Q. (2022). Cathepsin B deteriorates diabetic cardiomyopathy induced by streptozotocin via promoting NLRP3-mediated pyroptosis. Mol. Ther. Nucleic Acids.

[76] Meng L., Lin H., Huang X., Weng J., Peng F., Wu S. (2022). METTL14 suppresses pyroptosis and diabetic cardiomyopathy by downregulating TINCR lncRNA. Cell Death Dis..

[77] Yao J., Li Y., Jin Y., Chen Y., Tian L., He W. (2021). Synergistic cardioptotection by tilianin and syringin in diabetic cardiomyopathy involves interaction of TLR4/NF-κB/NLRP3 and PGC1a/SIRT3 pathways. Int. Immunopharmacol..

[78] Wei C., Xu J., Liu Y., Qadir J., Zhang S., Yuan H. (2023). Exogenous Spermidine Alleviates Diabetic Myocardial Fibrosis Via Suppressing Inflammation and Pyroptosis in db/db Mice. Balk. Med. J..

[79] Begum R., Thota S., Abdulkadir A., Kaur G., Bagam P., Batra S. (2022). NADPH oxidase family proteins: Signaling dynamics to disease management. Cell Mol. Immunol..

[80] Devant P., Boršić E., Ngwa E.M., Xiao H., Chouchani E.T., Thiagarajah J.R., Hafner-Bratkovič I., Evavold C.L., Kagan J.C. (2023). Gasdermin D pore-forming activity is redox-sensitive. Cell Rep..

[81] Wei H., Bu R., Yang Q., Jia J., Li T., Wang Q., Chen Y. (2019). Exendin-4 Protects against Hyperglycemia-Induced Cardiomyocyte Pyroptosis via the AMPK-TXNIP Pathway. J. Diabetes Res..

[82] Peng M.-L., Fu Y., Wu C.-W., Zhang Y., Ren H., Zhou S.-S. (2022). Signaling Pathways Related to Oxidative Stress in Diabetic Cardiomyopathy. Front. Endocrinol..

[83] Evavold C.L., Hafner-Bratkovič I., Devant P., D’Andrea J.M., Ngwa E.M., Boršić E., Doench J.G., LaFleur M.W., Sharpe A.H., Thiagarajah J.R. (2021). Control of gasdermin D oligomerization and pyroptosis by the Ragulator-Rag-mTORC1 pathway. Cell.

[84] Wang B., Yin Q. (2017). AIM2 inflammasome activation and regulation: A structural perspective. J. Struct. Biol..

[85] Wang X., Pan J., Liu H., Zhang M., Liu D., Lu L., Tian J., Liu M., Jin T., An F. (2019). AIM2 gene silencing attenuates diabetic cardiomyopathy in type 2 diabetic rat model. Life Sci..

[86] Wang Y., Chen C., Chen J., Sang T., Peng H., Lin X., Zhao Q., Chen S., Eling T., Wang X. (2022). Overexpression of NAG-1/GDF15 prevents hepatic steatosis through inhibiting oxidative stress-mediated dsDNA release and AIM2 inflammasome activation. Redox Biol..

[87] Nie L., Zhao P., Yue Z., Zhang P., Ji N., Chen Q., Wang Q. (2021). Diabetes induces macrophage dysfunction through cytoplasmic dsDNA/AIM2 associated pyroptosis. J. Leukoc. Biol..

[88] Shimada K., Crother T.R., Karlin J., Dagvadorj J., Chiba N., Chen S., Ramanujan V.K., Wolf A.J., Vergnes L., Ojcius D.M. (2012). Oxidized mitochondrial DNA activates the NLRP3 inflammasome during apoptosis. Immunity.

[89] Hu B., Jin C., Li H.-B., Tong J., Ouyang X., Cetinbas N.M., Zhu S., Strowig T., Lam F.C., Zhao C. (2016). The DNA-sensing AIM2 inflammasome controls radiation-induced cell death and tissue injury. Science.

[90] Fidler T.P., Xue C., Yalcinkaya M., Hardaway B., Abramowicz S., Xiao T., Liu W., Thomas D.G., Hajebrahimi M.A., Pircher J. (2021). The AIM2 inflammasome exacerbates atherosclerosis in clonal haematopoiesis. Nature.

[91] Yarovinsky T.O., Su M., Chen C., Xiang Y., Tang W.H., Hwa J. (2023). Pyroptosis in cardiovascular diseases: Pumping gasdermin on the fire. Semin. Immunol..

[92] Ma X.M., Geng K., Law B.Y.-K., Wang P., Pu Y.L., Chen Q., Xu H.W., Tan X.Z., Jiang Z.Z., Xu Y. (2023). Lipotoxicity-induced mtDNA release promotes diabetic cardiomyopathy by activating the cGAS-STING pathway in obesity-related diabetes. Cell Biol. Toxicol..

[93] Han J., Dai S., Zhong L., Shi X., Fan X., Zhong X., Lin W., Su L., Lin S., Han B. (2022). GSDMD (Gasdermin D) Mediates Pathological Cardiac Hypertrophy and Generates a Feed-Forward Amplification Cascade via Mitochondria-STING (Stimulator of Interferon Genes) Axis. Hypertension.

[94] You J., Li X., Dai F., Liu J., Zhang Q., Guo W. (2023). GSDMD-mediated pyroptosis promotes cardiac remodeling in pressure overload. Clin. Exp. Hypertens..

[95] Zhang Y.-F., Zhou L., Mao H.-Q., Yang F.-H., Chen Z., Zhang L. (2021). Mitochondrial DNA leakage exacerbates odontoblast inflammation through gasdermin D-mediated pyroptosis. Cell Death Discov..

[96] Fu L., Zhang J., Lin Z., Li Y., Qin G. (2022). CircularRNA circ_0071269 knockdown protects against from diabetic cardiomyopathy injury by microRNA-145/gasdermin A axis. Bioengineered.

[97] Yuan Q., Sun Y., Yang F., Yan D., Shen M., Jin Z., Zhan L., Liu G., Yang L., Zhou Q. (2023). CircRNA DICAR as a novel endogenous regulator for diabetic cardiomyopathy and diabetic pyroptosis of cardiomyocytes. Signal Transduct. Target. Ther..

[98] Yang F., Qin Y., Lv J., Wang Y., Che H., Chen X., Jiang Y., Li A., Sun X., Yue E. (2018). Silencing long non-coding RNA Kcnq1ot1 alleviates pyroptosis and fibrosis in diabetic cardiomyopathy. Cell Death Dis..

[99] Xiao W., Zheng D., Chen X., Yu B., Deng K., Ma J., Wen X., Hu Y., Hou J. (2021). Long non-coding RNA MIAT is involved in the regulation of pyroptosis in diabetic cardiomyopathy via targeting miR-214-3p. iScience.

[100] Wu A., Sun W., Mou F. (2021). lncRNA-MALAT1 promotes high glucose-induced H9C2 cardiomyocyte pyroptosis by downregulating miR-141-3p expression. Mol. Med. Rep..

[101] Abo-Saif M.A., Ragab A.E., Ibrahim A.O., Abdelzaher O.F., Mehanyd A.B.M., Saber-Ayad M., El-Feky O.A. (2023). Pomegranate peel extract protects against the development of diabetic cardiomyopathy in rats by inhibiting pyroptosis and downregulating LncRNA-MALAT1. Front. Pharmacol..

[102] Song S., Ding Y., Dai G.-L., Zhang Y., Xu M.-T., Shen J.-R., Chen T.-T., Chen Y., Meng G.-L. (2021). Sirtuin 3 deficiency exacerbates diabetic cardiomyopathy via necroptosis enhancement and NLRP3 activation. Acta Pharmacol. Sin..

[103] Xiong J., Zhou Q. (2023). The lncRNA HOTAIR attenuates pyroptosis of diabetic cardiomyocytes by recruiting FUS to regulate SIRT3 expression. Kaohsiung J. Med. Sci..

[104] Liu J., Sun M., Wang J., Sun Z., Wang G. (2023). HOTAIR regulates SIRT3-mediated cardiomyocyte survival after myocardial ischemia/reperfusion by interacting with FUS. BMC Cardiovasc. Disord..

[105] Tang G., Duan F., Li W., Wang Y., Zeng C., Hu J., Li H., Zhang X., Chen Y., Tan H. (2019). Metformin inhibited Nod-like receptor protein 3 inflammasomes activation and suppressed diabetes-accelerated atherosclerosis in apoE^−/−^ mice. Biomed. Pharmacother..

[106] Yang F., Qin Y., Wang Y., Meng S., Xian H., Che H., Lv J., Li Y., Yu Y., Bai Y. (2019). Metformin Inhibits the NLRP3 Inflammasome via AMPK/mTOR-dependent Effects in Diabetic Cardiomyopathy. Int. J. Biol. Sci..

[107] Zou R., Nie C., Pan S., Wang B., Hong X., Xi S., Bai J., Yu M., Liu J., Yang W. (2022). Co-administration of hydrogen and metformin exerts cardioprotective effects by inhibiting pyroptosis and fibrosis in diabetic cardiomyopathy. Free Radic Biol. Med..

[108] Liu B., Xu J., Lu L., Gao L., Zhu S., Sui Y., Cao T., Yang T. (2023). Metformin induces pyroptosis in leptin receptor-defective hepatocytes via overactivation of the AMPK axis. Cell Death Dis..

[109] Chen X., Ren L., Liu X., Sun X., Dong C., Jiang Y., Qin Y., Qu H., Jiao J., Wang S. (2020). Ranolazine protects against diabetic cardiomyopathy by activating the NOTCH1/NRG1 pathway. Life Sci..

[110] Ren L., Chen X., Nie B., Qu H., Ju J., Bai Y. (2022). Ranolazine Inhibits Pyroptosis via Regulation of miR-135b in the Treatment of Diabetic Cardiac Fibrosis. Front. Mol. Biosci..

[111] Kim S.R., Lee S.-G., Kim S.H., Kim J.H., Choi E., Cho W., Rim J.H., Hwang I., Lee C.J., Lee M. (2020). SGLT2 inhibition modulates NLRP3 inflammasome activity via ketones and insulin in diabetes with cardiovascular disease. Nat. Commun..

[112] Biegus J., Voors A.A., Collins S.P., Kosiborod M.N., Teerlink J.R., Angermann C.E., Tromp J., Ferreira J.P., Nassif M.E., Psotka M.A. (2023). Impact of empagliflozin on decongestion in acute heart failure: The EMPULSE trial. Eur. Heart J..

[113] Voors A.A., Angermann C.E., Teerlink J.R., Collins S.P., Kosiborod M., Biegus J., Ferreira J.P., Nassif M.E., Psotka M.A., Tromp J. (2022). The SGLT2 inhibitor empagliflozin in patients hospitalized for acute heart failure: A multinational randomized trial. Nat. Med..

[114] Liu P., Zhang Z., Wang J., Zhang X., Yu X., Li Y. (2021). Empagliflozin protects diabetic pancreatic tissue from damage by inhibiting the activation of the NLRP3/caspase-1/GSDMD pathway in pancreatic β cells: In vitro and in vivo studies. Bioengineered.

[115] Madonna R., Moscato S., Cufaro M.C., Pieragostino D., Mattii L., Del Boccio P., Ghelardoni S., Zucchi R., De Caterina R. (2023). Empagliflozin inhibits excessive autophagy through the AMPK/GSK3β signalling pathway in diabetic cardiomyopathy. Cardiovasc. Res..

[116] Martín-Sánchez F., Diamond C., Zeitler M., Gomez A.I., Baroja-Mazo A., Bagnall J., Spiller D., White M., Daniels M.J.D., Mortellaro A. (2016). Inflammasome-dependent IL-1β release depends upon membrane permeabilisation. Cell Death Differ..

[117] Rathkey J.K., Zhao J., Liu Z., Chen Y., Yang J., Kondolf H.C., Benson B.L., Chirieleison S.M., Huang A.Y., Dubyak G.R. (2018). Chemical disruption of the pyroptotic pore-forming protein gasdermin D inhibits inflammatory cell death and sepsis. Sci. Immunol..

[118] Li Y., Tu Z., Chen F., Yang X., Deng R., Su F., Cheng Z., Li S., Ong S.-B., Wang D. (2023). Anti-inflammatory effect of Danhong injection through inhibition of GSDMD-mediated pyroptosis. Phytomedicine.

[119] Humphries F., Shmuel-Galia L., Ketelut-Carneiro N., Li S., Wang B., Nemmara V.V., Wilson R., Jiang Z., Khalighinejad F., Muneeruddin K. (2020). Succination inactivates gasdermin D and blocks pyroptosis. Science.

[120] Hu J.J., Liu X., Xia S., Zhang Z., Zhang Y., Zhao J., Ruan J., Luo X., Lou X., Bai Y. (2020). FDA-approved disulfiram inhibits pyroptosis by blocking gasdermin D pore formation. Nat. Immunol..

[121] Tang G., Li S., Zhang C., Chen H., Wang N., Feng Y. (2021). Clinical efficacies, underlying mechanisms and molecular targets of Chinese medicines for diabetic nephropathy treatment and management. Acta Pharm. Sin. B.

[122] Zhou J., Yan S., Guo X., Gao Y., Chen S., Li X., Zhang Y., Wang Q., Zheng T., Chen L. (2023). Salidroside protects pancreatic β-cells against pyroptosis by regulating the NLRP3/GSDMD pathway in diabetic conditions. Int. Immunopharmacol..

[123] Han J., Zuo Z., Shi X., Zhang Y., Peng Z., Xing Y., Pang X. (2023). Hirudin ameliorates diabetic nephropathy by inhibiting Gsdmd-mediated pyroptosis. Cell Biol. Toxicol..

[124] Zhu J., Chen H., Le Y., Guo J., Liu Z., Dou X., Lu D. (2022). Salvianolic acid A regulates pyroptosis of endothelial cells via directly targeting PKM2 and ameliorates diabetic atherosclerosis. Front. Pharmacol..

[125] Sun S., Dawuti A., Gong D., Wang R., Yuan T., Wang S., Xing C., Lu Y., Du G., Fang L. (2022). Puerarin-V Improve Mitochondrial Respiration and Cardiac Function in a Rat Model of Diabetic Cardiomyopathy via Inhibiting Pyroptosis Pathway through P2X7 Receptors. Int. J. Mol. Sci..

[126] Zhang H., Chen X., Zong B., Yuan H., Wang Z., Wei Y., Wang X., Liu G., Zhang J., Li S. (2018). Gypenosides improve diabetic cardiomyopathy by inhibiting ROS-mediated NLRP3 inflammasome activation. J. Cell Mol. Med..

[127] Yang L., Cheng C.-F., Li Z.-F., Huang X.-J., Cai S.-Q., Ye S.-Y., Zhao L.-J., Xiong Y., Chen D.-F., Liu H.-L. (2023). Berberine blocks inflammasome activation and alleviates diabetic cardiomyopathy via the miR-18a-3p/Gsdmd pathway. Int. J. Mol. Med..

[128] Gao G., Fu L., Xu Y., Tao L., Guo T., Fang G., Zhang G., Wang S., Qin T., Luo P. (2022). Cyclovirobuxine D Ameliorates Experimental Diabetic Cardiomyopathy by Inhibiting Cardiomyocyte Pyroptosis via NLRP3 in vivo and in vitro. Front. Pharmacol..

[129] Jiang K., Tu Z., Chen K., Xu Y., Chen F., Xu S., Shi T., Qian J., Shen L., Hwa J. (2022). Gasdermin D inhibition confers antineutrophil-mediated cardioprotection in acute myocardial infarction. J. Clin. Investig..

[130] Sun S., Gong D., Liu R., Wang R., Chen D., Yuan T., Wang S., Xing C., Lv Y., Du G. (2023). Puerarin Inhibits NLRP3-Caspase-1-GSDMD-Mediated Pyroptosis via P2X7 Receptor in Cardiomyocytes and Macrophages. Int. J. Mol. Sci..

